# RFRP Neurons – The Doorway to Understanding Seasonal Reproduction in Mammals

**DOI:** 10.3389/fendo.2016.00036

**Published:** 2016-05-03

**Authors:** Jo B. Henningsen, François Gauer, Valérie Simonneaux

**Affiliations:** ^1^Institut des Neurosciences Cellulaires et Intégratives, Centre national de la recherche scientifique (CNRS), University of Strasbourg, Strasbourg, France

**Keywords:** RFRP, mediobasal hypothalamus, melatonin, seasonal reproduction, photoperiod, TSH

## Abstract

Seasonal control of reproduction is critical for the perpetuation of species living in temperate zones that display major changes in climatic environment and availability of food resources. In mammals, seasonal cues are mainly provided by the annual change in the 24-h light/dark ratio (i.e., photoperiod), which is translated into the nocturnal production of the pineal hormone melatonin. The annual rhythm in this melatonin signal acts as a synchronizer ensuring that breeding occurs when environmental conditions favor survival of the offspring. Although specific mechanisms might vary among seasonal species, the hypothalamic RF (Arg–Phe) amide-related peptides (RFRP-1 and -3) are believed to play a critical role in the central control of seasonal reproduction and in all seasonal species investigated, the RFRP system is persistently inhibited in short photoperiod. Central chronic administration of RFRP-3 in short day-adapted male Syrian hamsters fully reactivates the reproductive axis despite photoinhibitory conditions, which highlights the importance of the seasonal changes in RFRP expression for proper regulation of the reproductive axis. The acute effects of RFRP peptides, however, depend on species and photoperiod, and recent studies point toward a different role of RFRP in regulating female reproductive activity. In this review, we summarize the recent advances made to understand the role and underlying mechanisms of RFRP in the seasonal control of reproduction, primarily focusing on mammalian species.

## Introduction

Animals living in temperate and boreal latitudes experience marked seasonal changes in their environment. To overcome these environmental changes, thus increasing their chances of survival, they show seasonal changes in several aspects of their physiology, i.e., reproduction, metabolism, and behavior. Annual variations in day length are translated into an endocrine message, namely, the nocturnal secretion of the pineal hormone melatonin, which acts as a potent seasonal synchronizer of biological functions, especially reproductive activity (1–4).

The effects of photoperiod on reproductive function have long been established, and recent studies have made much progress in describing key components and pathways involved in this adaptive process. The RFamide peptide kisspeptin (kp), a very potent stimulator of gonadotropin-releasing hormone (GnRH) release, shows photoperiodic variations and was therefore thought to be a likely candidate for the photoperiodic control of reproduction in seasonal breeders, but increasing evidence now points toward another RFamide peptide, the RF (Arg-Phe) amide-related peptide (RFRP) as the critical intermediate between the melatonin-dependent photoperiodic signal and central control of the reproductive axis (5–8).

Herein, we review our current understanding of the RFRP system in seasonal breeders, reporting the mechanisms through which melatonin impacts on RFRP synthesis and the effects of RFRPs in the seasonal control of reproduction including species-dependant and sex-specific variations in the RFRP system.

## The Pineal Hormone Melatonin Synchronizes Reproduction with Seasons

In seasonal species, reproductive activity is restricted to a particular time of the year so that birth occurs when warmer temperatures and better accessibility to food increase the chances of survival of the offspring. Early studies have demonstrated that synchronization of reproductive activity with season is driven by the pineal hormone melatonin (2). Photic information reaches the pineal gland *via* the retino–hypothalamo–pineal pathway that during nighttime generates a release of norepinephrine, which acts as a potent and reliable regulator of the rhythmic release of melatonin from the pineal gland. As a consequence, melatonin is synthesized and secreted in a diurnal fashion with a dramatic increase during nighttime that returns to nearly undetectable levels at daytime with the duration of elevated melatonin depending on night length (1–4, 8, 9). In long photoperiod (LP) breeders, i.e., the Syrian hamster (*Mesocricetus auratus*) that is widely used as a rodent model to study seasonal reproduction, short day lengths represented by a long nocturnal duration of melatonin secretion inhibits the reproductive axis, and removal of the melatonin signal by pinealectomy prevents this short day inhibition of reproductive activity (2). In contrast to small rodents, larger mammals with a longer gestation time, such as the sheep, are sexually active in short photoperiod (SP) and becomes sexually quiescent after transfer to LP conditions (10). Although the reproductive timing is opposite in hamsters and sheep, in both cases, the photoperiodic changes in circulating levels of melatonin synchronize reproduction with seasons.

### Melatonin Modes And Sites of Action on the Reproductive Axis

Three melatonin receptor subtypes have been characterized so far; MT1 (Mel1a), MT2 (Mel1b), and Mel1c with its mammalian ortholog GPR50 (11, 12). Using the highly specific 2-[^125^I]-iodomelatonin, high affinity melatonin-binding sites have been found in the hypothalamus and the *pars tuberalis* (PT) of mammals, and among species, the highest concentration of melatonin receptors is found in the PT (8, 13–16). The MT1 subtype seems to be dominantly expressed throughout species and is known to be responsible for the neuroendocrine integration of season (17, 18). Indeed, Siberian hamsters (also known as the Djungarian hamster or *Phodopus sungorus*) show seasonal reproductive responses to melatonin despite lacking a functional MT2 receptor (19). Maywood et al. found that site specific lesions of iodomelatonin-binding sites in the mediobasal hypothalamus (MBH) prevent testicular regression in Syrian hamsters exposed to SP (20) and in Siberian hamsters, melatonin infusion into or lesions of the suprachiasmatic nucleus (SCN), alter the reproductive response to seasonal changes (21, 22). Finally, in sheep, the premammilary region of the hypothalamus contains melatonin-binding sites (23, 24) and melatonin implantations in the area of this structure, but not the PT, were shown to prevent synchronization of reproduction with photoperiod (24–27). Altogether, these data have pointed toward the potential importance of these hypothalamic regions for proper integration of the melatonin-dependent photoperiodic signal onto the reproductive axis. However, it has not been possible to determine whether and how melatonin would act directly on these hypothalamic sites. Although a direct hypothalamic effect of melatonin cannot be excluded, accumulating evidence now points toward the PT as the major site for the hypothalamic integration of the melatonin signal in seasonal breeders (8, 28–30).

## TSH, Thyroid Hormones, and the Melatonin-Driven Reproductive Activity

In 2003, Yoshimura and colleagues made a remarkable finding that unveiled a link between the thyroid-stimulating hormone (TSH) pathway and seasonal reproduction. They showed that light-induced type 2 thyroid hormone deiodinase (Dio2) expression in the MBH and subsequent hormone conversion of thyroxine (T4) into the bioactive triiodothyronine (T3) regulate the photoperiodic response of gonads in birds (31). Since this discovery, it has been shown that TSH expression in the PT is regulated by photoperiod in a melatonin dependent manner and that TSH stimulates Dio2 expression in seasonal mammals (29, 32, 33). MT1-expressing cells in the PT synthesize TSH, and its production in the PT is strongly inhibited by the SP pattern of melatonin (34–36). Recent work moreover disclosed that melatonin regulates the photoperiodic changes in TSH expression in the PT *via* differential effects on clock gene expression and on the transcription of the co-activator EYA3 (37–39). Another primary response to photoperiodic changes in melatonin is the opposite regulation of Dio2 and type 3 thyroid hormone deiodinase (Dio3) expression in the MBH (29, 31, 33, 40–43). While Dio2 catalyzes the conversion of T4 into T3, Dio3 catalyzes the conversion of T3 to the biological inactive T2. Thus, in concert, Dio2 and Dio3 regulate the hypothalamic T4/T3 balance according to photoperiod, with a higher production of T3 in LP as compared to SP (5, 43–45). Thyroid hormones have long been known to be important for the transitions between the breeding and non-breeding states, i.e., in 1940s, thyroidectomy in starlings was reported to result in persistent breeding (46). Similarly, thyroidectomized sheep remain in the breeding state when changing from spring to anestrous and T4 replacement can reverse these effects, but interestingly, thyroidectomy displays no effect in the transition from the anestrous state to the breeding state (47–50). Moreover, T3 administration in the LP-breeding Siberian hamster blocks the SP-induced gonadal regression (44, 51, 52).

**Figure 1:**
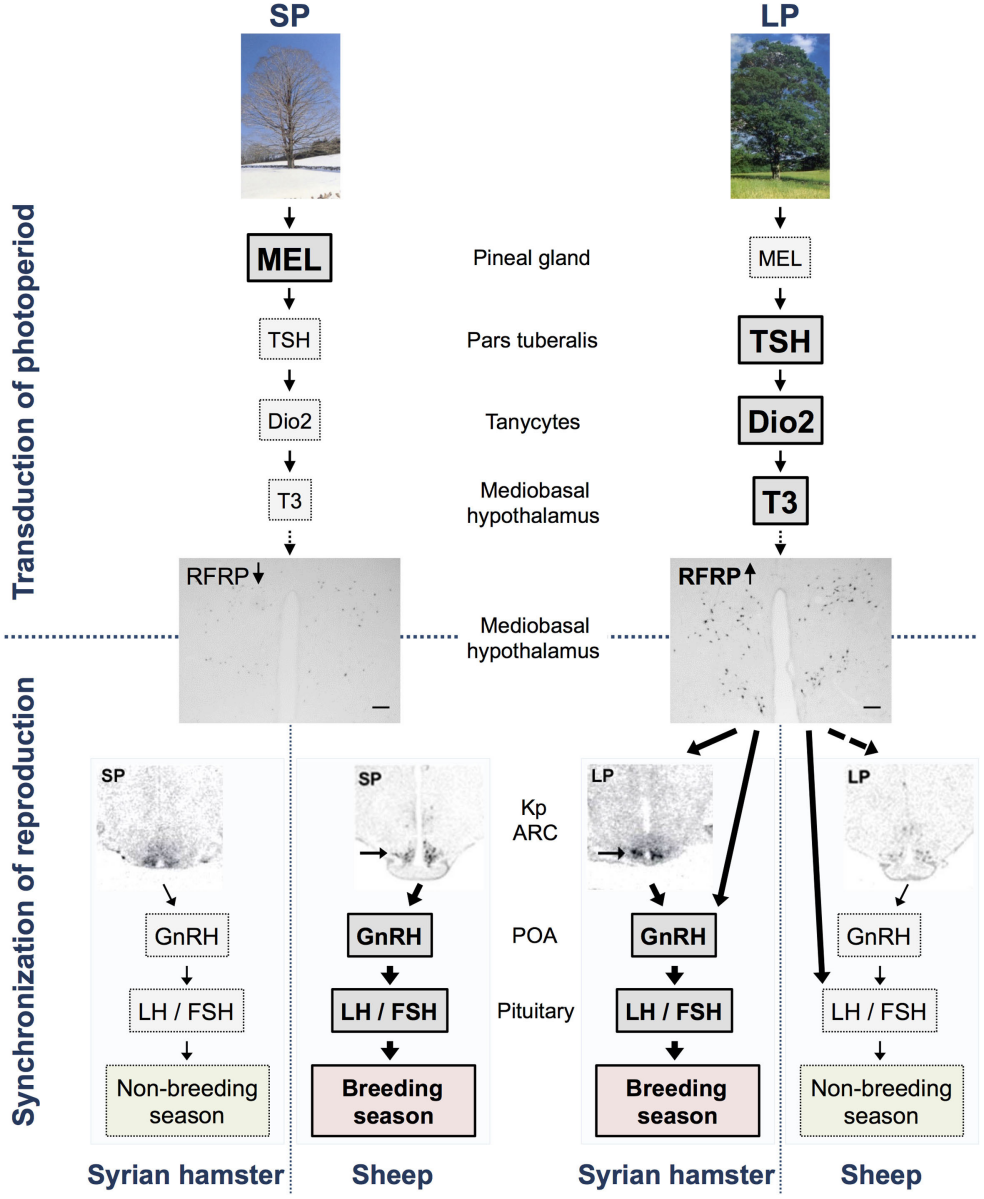
**Model of the transduction of photoperiod and seasonal regulation of the reproductive axis in long (Syrian hamsters) and short day (sheep) breeders**. In short photoperiod (SP), the large production of melatonin from the pineal gland inhibits TSH synthesis in the *pars tuberalis*, whereas the lower production of melatonin in long photoperiod (LP) allows the synthesis and release of TSH. TSH is transmitted *via* TSH receptors expressed in tanycytes surrounding the third ventricle and activates the enzyme deiodinase 2 (Dio2). Dio2 ultimately controls and increases the local availability of the active form of the thyroid hormone, T3, in the mediobasal hypothalamus. Subsequently, T3 regulates the expression of RFRP also in the mediobasal hypothalamus so that there is a high expression in LP and a low expression in SP in both LP and SP breeders, as demonstrated with pictures of RFRP-ir neurons in brains from Syrian hamster kept in LP and SP [scale bar 100 μm, taken from Ref. (59)]. In Syrian hamsters, RFRP subsequently acts either directly on GnRH neurons or indirectly *via* kisspeptin (kp) neurons (indicated by arrows) or other interneurons in the arcuate nucleus (ARC) to synchronize reproduction with season. In sheep, RFRP regulates the reproductive axis directly at the level of the pituitary (indicated by arrow) and possibly also directly or indirectly *via* kp neurons (indicated by dotted arrow) and/or GnRH neurons. Expression of the gene encoding kp in the ARC displays an opposite photoperiodic regulation in the two species being elevated in both LP-adapted sexually active Syrian hamsters [see arrow in picture taken from Ref. (33, 60)] and SP-adapted sexually active sheep [see arrow in picture taken from Ref. (61)]. This model does not describe specific effects of RFRP-3 reported in each species, sex, and photoperiod, and readers are referred to Table 1 for a detailed summary.

Both deiodinases are highly expressed in a population of specialized glial cells in the ependymal cell layer lining the third ventricle named tanycytes (53). Interestingly, tanycytes co-express the TSH receptor (TSHR), and recent data clearly show that activation of these receptors increases *Dio2* expression, thereby increasing levels of T3 in the MBH, in a number of seasonal mammals (5, 29, 33, 43, 54–57). In line with these observations, a recent study shows that PT-derived TSH, in contrast to *pars distalis*-derived TSH, does not stimulate the thyroid gland, but rather acts *via* TSHR on the tanycytes (58). Altogether, these studies have unveiled a conserved photoperiodic transduction pathway explaining how the melatonin signal is integrated in the PT and transduced into a local thyroid message in the MBH (8) (Figure 1).

Thyroid hormones mediate their neuroendocrine effects through still undefined hypothalamic sites since no cellular phenotyping of their receptors have been reported in the hypothalamus. Interestingly, however, a recent study found that chronic TSH administration in SP-adapted Siberian and Syrian male hamsters reactivates the reproductive axis, while at the same time increasing the expression of two known hypothalamic regulators of reproductive output, RFRP and Kp, suggesting that the melatonin signal reaches the reproductive axis *via* the TSH/thyroid hormone pathway acting on these neurons (5) rather than *via* direct hypothalamic effect as previously suggested (20).

## Hypothalamic Regulation of Seasonal Reproduction

Hypothalamic control of the reproductive axis is commonly regulated among species through the release of GnRH from GnRH fiber terminals projecting to the median eminence. GnRH is released into the portal blood system from which it regulates the synthesis and release of pituitary gonadotropins. Despite the marked decrease of GnRH release during sexual quiescence, most seasonal species display an unchanged number and level of GnRH neurons and GnRH-immunoreactivity (ir) in the different photoperiods (62, 63). GnRH synthesis and release is regulated upstream by various signals, especially two RF-amide peptides released from, respectively, RFRP neurons in and around the dorso/ventromedial hypothalamus (DMH/VMH) and kp neurons in the anteroventral periventricular nucleus (AVPV) and medial preoptic nucleus (MPN), and in the arcuate nucleus (ARC). Kp peptides are potent stimulators of the reproductive axis and acts directly on GnRH neurons through their cognate receptor GPR54 (64, 65). Interestingly, kp expression both in the MPN/AVPV and ARC was found to be significantly downregulated by melatonin in SP-adapted sexually inactive Syrian hamsters (60, 66). In the SP-breeding sheep, kp expression is oppositely upregulated in SP (61, 67, 68), suggesting that ARC kp expression reflects the breeding state rather than the seasonal state of the animal (Figure 1). In both hamsters and sheep, continuous infusion of kp during sexual quiescence fully restores reproductive activity (60, 69–71), and kp neurons are thus a pivotal component between the photoperiodic signal and seasonal activation of GnRH neurons. This statement is, however, complicated by results showing a lowered ARC kp expression in LP-breeding Siberian (72, 73) and European (74) hamsters as compared to SP. While these differences in the photoperiodic regulation of ARC kp might be explained by different feedback mechanisms of sex steroids, it also suggests that these neurons are differently implicated in the photoperiodic control of reproduction from one species to another. Therefore, it seems unlikely that kp neurons are solely responsible for mediating the melatonin-dependent seasonal signal onto the reproductive axis. By contrast, increasing results demonstrate that the photoperiodic regulation of RFRP expression within the DMH/VMH is conserved among seasonal species (75), suggesting that RFRP neurons may be potential candidates for integration of the photoperiodic signal.

## The RFRP System

RF (Arg–Phe) amide-related peptides were discovered in birds and mammals in 2000 and found to be primarily expressed in neurons located in the paraventricular nucleus (PVN) and in between the DMH and VMH in birds and rats, respectively (76, 77). In birds, the peptide was shown to inhibit gonadotropin secretion from cultured quail pituitaries and thus termed gonadotropin-inhibitory hormone (GnIH) (76). The avian GnIH precursor encodes one GnIH and two GnIH-related peptides (GnIH-RP-1 and GnIH-RP-2). GnIH and GnIH-RP-1 contain an LPLRFa motif in the C-teminal, whereas GnIH-RP-2 contains an LPQRFa motif. The mammalian gene named *RFamide-related peptide* (*Rfrp*) encodes RFRP-1, -2, and -3 peptides, of which RFRP-1 (containing an LPLRFa motif) and RFRP-3 (containing a C-terminal LPQRFa motif) are functional peptides (77–79). RFRP-3 and GnIH have been shown to inhibit GnRH neuron activity and gonadotropin release in several seasonal (sheep, hamster, and quail) and non-seasonal (rat and mouse) species (76, 80–87). Moreover, there is evidence of a hypophysiotropic effect of GnIH and RFRP-3 in birds and ewes, respectively, although the effect in ewe is still of controversy since in one study intravenous (iv) infusion of RFRP-3 inhibits pulsatile LH secretion in ovariectomized ewes (81), whereas two other studies find no variation in LH plasma concentrations in neither ovariectomized nor intact ewes injected either intracerebroventricular (icv) or iv with RFRP-3 (88, 89). Two recent studies reported a stimulatory effect of central administration of RFRP-3 in male Syrian and Siberian hamster, indicating that the effects of the peptide are species dependent (6, 7). Strikingly, while RFRP-3 activates the reproductive axis in male Syrian hamsters (6), the avian GnIH inhibits LH secretion in ovariectomized females (84), adding a supplementary sex difference in the effects of the peptides.

Recently, a study of Tena-Sempere and colleagues (86) reported that mouse KO for GPR147, the cognate receptor for RFRPs, does not display strong reproductive phenotypic alterations as compared to wild-type mice. Moderate changes are, however, observed in GPR147-deficient mice, as during pubertal transition, male KO mice exhibit increased LH levels, and in adulthood, FSH levels are higher in both female and male KO mice as compared to wild-type mice. Moreover, litter sizes from KO mice are slightly increased as compared to wild-type litter sizes. Interestingly, the male KO mice moreover show an increased level of kp expression in the ARC, but not in the MPN/AVPV, suggesting that in male mice, RFRP neurons provide a tonic inhibition on ARC kp neurons.

### RFRP Modes and Sites of Action

RFRP neurons are mainly found in and around the DMH/VMH from where they project to multiple regions of the rodent brain. RFRP-ir fibers are found especially in the preoptic area/organum vasculosum of the lamina terminalis (POA/OVLT), MPN/AVPV, the anterior part of the SCN, PVN, anterior hypothalamus, VMH, and ARC as well as in the bed nucleus of the stria terminalis, habenular nuclei, and PVN of the thalamus (7, 59, 84). RFRP terminals make apparent contact to 20–40% of GnRH somas in rodents and sheep (7, 67, 84, 90), and in female mice, around 20% of MPN/AVPV kp neurons and 35% of ARC kp neurons receive RFRP fiber contacts (90, 91). In mice, RFRP-3 application to brain slices inhibits the firing rate of 41% GnRH neurons and stimulates the firing rate of 12% of the GnRH neurons (82), whereas in male Syrian hamster, icv infusion of RFRP-3 induces c-FOS expression in 20–30% GnRH neurons but also in non-kisspeptinergic neurons of the ARC (6).

RFRPs bind preferentially to the G-protein-coupled receptor, GPR147 (also known as NPFF1). GPR147 has been found to couple to both stimulatory and inhibitory G protein subunits *in vitro* (92, 93), and in GPR147-transfected CHO cells, hRFRP-1 induces a maximal inhibition of a forskolin-induced cAMP accumulation, indicating that RFRP-1 might inhibit adenylate cyclase through a Gαi-bound receptor complex (94). However, the exact signaling events occurring downstream of GPR147 in its natural cellular environment still remains unknown. GPR147-encoding mRNA is widely distributed in the brain; however, particular strong expression is observed in hypothalamic regions as the POA/OVLT, MPN/AVPV, SCN, PVN, anterior hypothalamus, VMH, and ARC, and outside the hypothalamus in the posterior part of the bed nucleus of the stria terminalis, habenular nuclei, and the pyramidal cell layer of the hippocampus (59, 95, 96). Interestingly, GPR147 has been shown to be expressed in 15–33% of mice GnRH neurons and a subpopulation of kp neurons in the AVPV (5–16%) and the ARC (25%) (90, 91, 97). Altogether, these studies suggest that RFRPs can act directly on these central neuroendocrine regulators of reproduction.

## RFRP: A Critical Switch between Melatonin and the Reproductive Axis

### GnIH and Avian Seasonal Reproduction

Gonadotropin-inhibitory hormone inhibits LH release from cultured quail pituitaries (76), and to further support a direct pituitary effect of GnIH in quail, GnIH-ir fibers have been found to project to the median eminence in this species (98). Also in quail, studies have revealed that GnIH expression and release is directly regulated by melatonin acting on Mel1c receptors specifically expressed in GnIH neurons (99). In contrast to mammalian seasonal species, GnIH expression is increased by melatonin and consequently GnIH-ir expression is increased in SP as compared to LP (99). *In vitro* studies furthermore show that GnIH release has a diurnal rhythm and is increased during nighttime in quail hypothalamic explants (100). In house and song sparrows, GnIH-ir neurons are reported bigger toward the end of the breeding season (101). By contrast in wild Australia zebra finches, there is no variation in neither GnIH-ir nor *GnIH* expression between the breeding and non-breeding states (102).

### RFRP in Seasonal Mammalian Species

#### Photoperiodic Variations in the RFRP System

There are no circadian or day-to-night variations in RFRP mRNA expression in male Syrian (103) and European (74) hamsters, respectively. RFRP expression is however strongly regulated by photoperiod and is downregulated in SP in several seasonal breeders (6, 67, 74, 75, 103, 104). Studies in male Syrian and Siberian hamsters show the SP downregulation to be melatonin dependent (7, 103). Recent findings have revealed that in female Syrian hamsters as well, RFRP expression is downregulated in SP, probably driven by the same mechanisms (59). Interestingly, RFRP expression is similarly downregulated in SP in short-day breeders such as the sheep (67, 96). This suggests that the SP pattern of circulating melatonin displays a conserved inhibition on RFRP expression independently of whether mammals are long- or short-day breeders (Figure 1). Importantly, the photoperiodic/melatonin regulation of RFRP expression, in contrast to kp, may not be modulated by the gonadal hormone feed back because, although RFRP neurons express sex steroid receptors (84), neither gonadectomy nor sex-hormone implants alter RFRP expression in male (103) and female (Henningsen et al., unpublished) Syrian hamsters.

We have recently shown that GPR147 mRNA levels in the Syrian hamster’s brain also depends on photoperiod being downregulated in SP condition, and interestingly, this downregulation is much stronger and consistent in females as compared to males (59). In Siberian hamsters and sheep, the amount of GnRH neurons receiving RFRP fiber contacts is decreased in SP conditions (7, 81); however, in the Syrian hamster, we did not find any photoperiodic variations in numbers of RFRP-ir fibers projecting specifically to the OVLT or the ARC (59).

#### Species-Specific Differences in the Effects of RFRP on Seasonal Reproduction

We were the first to show that in male Syrian hamster, RFRP-3 is capable of stimulating the reproductive axis (6). In more details, RFRP-3 was found to stimulate GnRH neuronal activity, LH and FSH release, and testosterone production independently of the photoperiodic condition, although to a lesser extent in SP animals. Moreover, chronic central administration of RFRP-3 in SP-adapted male Syrian hamsters reactivated the reproductive axis *via* an increase in ARC kp expression, despite the animals being kept in SP-inhibitory conditions. The stimulatory effect of RFRP-3 observed in male Syrian hamsters fits well with the high RFRP expression in sexually active LP animals, and our data furthermore indicate that the stimulation of reproductive activity could be mediated *via* the ARC kp neurons. Thus, in the male Syrian hamster, RFRP neurons appear to integrate and transfer the seasonal input toward kp neurons. In another hamster species, the male Siberian hamster, RFRP-3 displays reverse effects depending on the photoperiodic condition, stimulating LH release in SP but decreasing LH levels in LP conditions (7). Although the mechanism underlying such photoperiod-dependent effect of RFRP-3 in this species is unknown, it might help to explain the upregulation of ARC kp in the sexually inactive SP-adapted Siberian hamsters (72, 105, 106). These observations indicate that in the two hamster species, RFRP-3 either has opposite sites of action in LP-adapted animals or is integrated differently along the hypothalamo–pituitary–gonadal axis.

Despite the controversies of RFRP’s effect in sheep, one study reported that iv administration of ovine RFRP-3 peptide (also referred to as GnIH3) inhibits LH release in ovariectomized ewe (107), which indicates that RFRPs might have a hypophysiotropic effect in ovine species, similarly to what is observed in avian species. Indeed, RFRP fibers have been shown to project to the median eminence, and RFRPs are detected in the portal blood of sheep (108, 109). As previous studies have reported, there is no evidence of peripheral effects of RFRPs on hamster’s reproduction (6), thus describing another fundamental difference in how the RFRP signal is integrated in the hypothalamo–pituitary axis among mammalian seasonal species. Interestingly, the opposite effects and photoperiodic regulation of RFRP between sheep and hamsters support our hypothesis that a similar neuroendocrine pathway is conserved between LP and SP breeders with the RFRPs playing a pivotal role in adapting reproductive activity to the environment (Figure 1). Further analyzes are required to test this hypothesis, in particular whether the inhibitory effect of RFRP-3 account for the lower expression of kp in LP-adapted sexually inactive sheep. The reported effects of RFRP-3 in seasonal mammals are summarized in Table 1.

**Table 1:** **Overview of the *in vivo* effects of RFRP-3 on reproduction in seasonal mammals**.

Species	Sex and status	Effects of RFRP-3	Reference
Siberian hamster	Male	Central administration inhibits LH release in LP	(7)
		Central administration stimulates LH release in SP	
Syrian hamster	Male	Central acute and continuous administration stimulates LH release in LP and SP. No peripheral effect	(6)
	Female OVX	Central and peripheral administration (GnIH) inhibits LH release in LP	(84)
Sheep	Female	Peripheral injection or a 4-h perfusion has no effect on kisspeptin-mediated increase in LH in LP	(89)
		Peripheral administration inhibits LH release in SP	(107)
	Female OVX	Repeated peripheral injection has no effect on pulsatile LH release in LP. 24-h perfusion has no effect on E2-induced LH surge in SP	(89)
		Central and peripheral administration has no effect on LH release in SP and LP	(88)
		Peripheral administration inhibits E2-induced LH surge in SP	(81)
		Peripheral administration inhibits pulsatile LH release in SP	(107)
		Peripheral administration inhibits GnRH-induced LH release. RFRP-3 is detected in the portal blood in SP and LP, with higher conc. detected in LP	(109)

A fascinating issue is to disclose how RFRPs can have opposite seasonal and species-dependent effects. As previously mentioned, GPR147 has been found to couple to both stimulatory and inhibitory G proteins *in vitro* (92, 93), but it remains to be established if there are fundamental species-specific differences in the downstream signaling cascades after activation of the GPR147. Alternatively, it is likely that the cellular response to RFRP is conserved among species, but mediated *via* different targets. In male Syrian hamsters, acute injections of RFRP-3 induce c-Fos expression in a subset of GnRH neurons but also in an unidentified population of neurons in the ARC (6), and one can speculate whether the observed stimulatory effect of RFRP-3 in the male Syrian, but also Siberian hamsters, arises through inhibition of inhibitory interneurons, thus resulting in the stimulatory outcome. Future studies should aim at phenotyping downstream targets of RFRP in order to understand better its various effects on the reproductive axis of seasonal breeders.

#### Sex Differences in Seasonal Rodents

In contrast to the stimulatory effect of RFRP-3, we have observed in the male Syrian hamsters (6), central injections of the avian RFRP, GnIH, inhibits LH release in ovariectomized females (84). It should be stressed that GnIH contains a -LPLRFa motif similar to that of the mammalian RFRP-1 and not RFRP-3. However, we recently found that central RFRP-3 administration in the intact female Syrian hamster inhibits LH release similarly to the effects observed with the GnIH ortholog (Henningsen et al., unpublished). These observations add supplementary sex-specific differences in the acute effects of RFRPs, at least in Syrian hamsters. In order to understand such opposite effect of RFRP-3, we explored other potential sex differences in the Syrian hamster RFRP system. We found that the number of RFRP neurons and the intensity of the immunoreactive labeling were markedly higher in females than in males adapted to LP conditions. In SP conditions however, RFRP expression is downregulated to a similar low level in both sexes (59). The number of RFRP-ir fibers projecting specifically to the MPN/AVPV is increased in SP as compared to LP in females, but not in males. Moreover, we found that the overall levels of GPR147 mRNA were higher in females than in males, regardless of photoperiod, and that the SP-induced downregulation of GPR147 mRNA levels were stronger in females than in males (59). A similar sex-specific difference in RFRP expression is also reported in the non-seasonal rat, where RFRP-1 expression is found to be higher in females as compared to males (110). Altogether, these findings point toward a particular importance of the RFRP system in seasonal as well as non-seasonal females.

### RFRP and Circadian Changes in Female Reproductive Activity

RFRP neurons project to the MPN/AVPV (59, 84, 90) where kp neurons provide the stimulatory signal onto the GnRH neurons causing the surge of LH and thereby ovulation (111). The surge of LH requests high circulating E2 levels, as an indicator of ovarian maturation, but its timing is also gated by a circadian signal to occur at the end of the resting period (112). It has been suggested that in female mammals, RFRP neurons mediate a SCN-generated circadian output onto the MPN/AVPV kp neurons, thereby modulating the timing and generation of the LH surge (113). Indeed, in the female Syrian hamster, a decrease in RFRP expression occurs around the time of the LH surge (113), and in rat and ewes, RFRP expression is similarly reduced during the preovulatory period (107, 110). Interestingly, a recent study showed that SCN-derived vasoactive intestinal peptide (VIP)-ergic terminal fibers projections are found in the area of where RFRP neurons are expressed in the female Syrian hamster and more importantly that central VIP administration markedly suppress RFRP cellular activity in the evening, but not in the morning (114). Altogether, these data point toward a specific circadian rhythm in RFRP expression and release in females, adding a supplementary role of RFRP in regulating reproductive activity in seasonal female species.

## Conclusion

Over the past nearly two decades, RFRPs have been extensively studied for their putative involvement in the regulation of the reproductive axis (87). Initially, RFRPs were thought to act with a similar inhibitory effect as the avian ortholog GnIH in all species and is still widely referred to as GnIH, despite its well-documented stimulatory effect in Siberian and Syrian hamster species (6, 7). These recent findings have challenged the conception of a conserved role of RFRPs throughout species and underline the necessity to delineate its effects in one species, sex, and physiological condition, such as season.

The marked downregulation of RFRP expression observed in seasonal mammals, independently of sex, species, and breeding behavior, provides evidence for a distinct and conserved role of this peptide in the integration of the photoperiodic melatonin signal *via* a TSH-dependent regulation of thyroid hormone locally in the MBH (5, 8, 75) (Figure 1).

In the Syrian hamster, RFRP has opposite effects in females and males, and there are strong sex-specific differences in the RFRP system manifested by a higher expression and stronger photoperiodic variations in the RFRP system in females as compared to males. Few studies have investigated the regulation and role of the RFRP system in female reproduction; however, findings so far strongly suggest that RFRP neurons play a critical role in female reproduction in regards to the timing of both daily and seasonal synchronization of their reproductive activity.

## Author Contributions

JH has written a large part of the review; FG and VS have supervised the writing, corrected the text, and helped in the design of the figure.

## Conflict of Interest Statement

The authors declare that the research was conducted in the absence of any commercial or financial relationships that could be construed as a potential conflict of interest.

## References

[1] Carter DS Goldman BD. Antigonadal effects of timed melatonin infusion in pinealectomized male Djungarian hamsters (*Phodopus sungorus* sungorus): duration is the critical parameter. Endocrinology (1983) 113:1261–7. 10.1210/endo-113-4-1261 6617572

[2] Hoffman RA Reiter RJ. Pineal gland: influence on gonads of male hamsters. Science (1965) 148:1609–11. 10.1126/science.148.3677.1609 14287606

[3] Malpaux B Migaud M Tricoire H Chemineau P. Biology of mammalian photoperiodism and the critical role of the pineal gland and melatonin. J Biol Rhythms (2001) 16:336–47. 10.1177/074873001129002051 11506379

[4] Pevet P. The role of the pineal gland in the photoperiodic control of reproduction in different hamster species. Reprod Nutr Dev (1988) 28:443–58. 10.1051/rnd:19880310 3045927

[5] Klosen P Sebert ME Rasri K Laran-Chich MP Simonneaux V. TSH restores a summer phenotype in photoinhibited mammals via the RF-amides RFRP3 and kisspeptin. FASEB J (2013) 27:2677–86. 10.1096/fj.13-229559 23538709

[6] Ancel C Bentsen AH Sebert ME Tena-Sempere M Mikkelsen JD Simonneaux V. Stimulatory effect of RFRP-3 on the gonadotrophic axis in the male Syrian hamster: the exception proves the rule. Endocrinology (2012) 153:1352–63. 10.1210/en.2011-1622 22275511

[7] Ubuka T Inoue K Fukuda Y Mizuno T Ukena K Kriegsfeld LJ Identification, expression, and physiological functions of Siberian hamster gonadotropin-inhibitory hormone. Endocrinology (2012) 153:373–85. 10.1210/en.2011-1110 22045661 PMC3249677

[8] Hazlerigg DG Simonneaux V Seasonal regulation of reproduction in mammals. In: Plant TM Zeleznik AJ, editors. Physiology of Reproduction. London: Elsevier Academic Press (2015). p. 1575–604.

[9] Karsch FJ Bittman EL Foster DL Goodman RL Legan SJ Robinson JE Neuroendocrine basis of seasonal reproduction. Recent Prog Horm Res (1984) 40:185–232. 6385166 10.1016/b978-0-12-571140-1.50010-4

[10] Bittman EL Karsch FJ. Nightly duration of pineal melatonin secretion determines the reproductive response to inhibitory day length in the ewe. Biol Reprod (1984) 30:585–93. 10.1095/biolreprod30.3.585 6722237

[11] Dufourny L Levasseur A Migaud M Callebaut I Pontarotti P Malpaux B GPR50 is the mammalian ortholog of Mel1c: evidence of rapid evolution in mammals. BMC Evol Biol (2008) 8:105. 10.1186/1471-2148-8-105 18400093 PMC2323367

[12] Reppert SM Weaver DR Godson C Melatonin receptors step into the light: cloning and classification of subtypes. Trends Pharmacol Sci (1996) 17:100–2. 10.1016/0165-6147(96)10005-5 8936344

[13] Weaver DR Rivkees SA Reppert SM. Localization and characterization of melatonin receptors in rodent brain by in vitro autoradiography. J Neurosci (1989) 9:2581–90. 2545841 10.1523/JNEUROSCI.09-07-02581.1989 PMC6569774

[14] Vanĕcek J Pavlík A Illnerová H. Hypothalamic melatonin receptor sites revealed by autoradiography. Brain Res (1987) 435:359–62. 10.1016/0006-8993(87)91625-8 2827856

[15] Vanĕcek J. Melatonin binding sites. J Neurochem (1988) 51:1436–40. 10.1111/j.1471-4159.1988.tb01108.x 3171587

[16] Williams LM Morgan PJ Hastings MH Lawson W Davidson G Howell HE. Melatonin receptor sites in the Syrian hamster brain and pituitary. Localization and characterization using [|]lodomelatonin*. J Neuroendocrinol (1989) 1:315–20. 10.1111/j.1365-2826.1989.tb00122.x 19210421

[17] Poirel V-J Cailotto C Streicher D Pévet P Masson-Pévet M Gauer F. MT1 melatonin receptor mRNA tissular localization by PCR amplification. Neuro Endocrinol Lett (2003) 24:33–8. 12743529

[18] Cogé F Guenin SP Fery I Migaud M Devavry S Slugocki C The end of a myth: cloning and characterization of the ovine melatonin MT(2) receptor. Br J Pharmacol (2009) 158:1248–62. 10.1111/j.1476-5381.2009.00453.x 19814723 PMC2782334

[19] Weaver DR Liu C Reppert SM Nature’s knockout: the Mel1b receptor is not necessary for reproductive and circadian responses to melatonin in Siberian hamsters. Mol Endocrinol (1996) 10:1478–87. 10.1210/mend.10.11.8923472 8923472

[20] Maywood ES Bittman EL Hastings MH. Lesions of the melatonin- and androgen-responsive tissue of the dorsomedial nucleus of the hypothalamus block the gonadal response of male Syrian hamsters to programmed infusions of melatonin. Biol Reprod (1996) 54:470–7. 10.1095/biolreprod54.2.470 8788201

[21] Bartness TJ Goldman BD Bittman EL. SCN lesions block responses to systemic melatonin infusions in Siberian hamsters. Am J Physiol (1991) 260:R102–12. 1899543 10.1152/ajpregu.1991.260.1.R102

[22] Badura LL Goldman BD. Central sites mediating reproductive responses to melatonin in juvenile male Siberian hamsters. Brain Res (1992) 598:98–106. 10.1016/0006-8993(92)90172-6 1486507

[23] de Reviers MM Ravault JP Tillet Y Pelletier J. Melatonin binding sites in the sheep pars tuberalis. Neurosci Lett (1989) 100:89–93. 10.1016/0304-3940(89)90665-4 2548131

[24] Malpaux B Daveau A Maurice-Mandon F Duarte G Chemineau P. Evidence that melatonin acts in the premammillary hypothalamic area to control reproduction in the ewe: presence of binding sites and stimulation of luteinizing hormone secretion by in situ microimplant delivery. Endocrinology (1998) 139:1508–16. 10.1210/endo.139.4.5879 9528928

[25] Lincoln GA Maeda KI. Reproductive effects of placing micro-implants of melatonin in the mediobasal hypothalamus and preoptic area in rams. J Endocrinol (1992) 132:201–15. 10.1677/joe.0.1320201 1541920

[26] Malpaux B Daveau A Maurice F Gayrard V Thiery JC. Short-day effects of melatonin on luteinizing hormone secretion in the ewe: evidence for central sites of action in the mediobasal hypothalamus. Biol Reprod (1993) 48:752–60. 10.1095/biolreprod48.4.752 8485239

[27] Malpaux B Skinner DC Maurice F. The ovine pars tuberalis does not appear to be targeted by melatonin to modulate luteinizing hormone secretion, but may be important for prolactin release. J Neuroendocrinol (1995) 7:199–206. 10.1111/j.1365-2826.1995.tb00748.x 7606246

[28] Johnston JD Ebling FJP Hazlerigg DG. Photoperiod regulates multiple gene expression in the suprachiasmatic nuclei and pars tuberalis of the Siberian hamster (*Phodopus sungorus*). Eur J Neurosci (2005) 21:2967–74. 10.1111/j.1460-9568.2005.04148.x 15978008

[29] Hanon EA Lincoln GA Fustin JM Dardente H Masson-Pevet M Morgan PJ Ancestral TSH mechanism signals summer in a photoperiodic mammal. Curr Biol (2008) 18:1147–52. 10.1016/j.cub.2008.06.076 18674911

[30] Dardente H Hazlerigg DG Ebling FJP. Thyroid hormone and seasonal rhythmicity. Front Endocrinol (2014) 5:19. 10.3389/fendo.2014.00019 24616714 PMC3935485

[31] Yoshimura T Yasuo S Watanabe M Iigo M Yamamura T Hirunagi K Light-induced hormone conversion of T4 to T3 regulates photoperiodic response of gonads in birds. Nature (2003) 426:178–81. 10.1038/nature02117 14614506

[32] Nakao N Ono H Yamamura T Anraku T Takagi T Higashi K Thyrotrophin in the pars tuberalis triggers photoperiodic response. Nature (2008) 452:317–22. 10.1038/nature06738 18354476

[33] Revel FG Saboureau M Pevet P Mikkelsen JD Simonneaux V Melatonin regulates type 2 deiodinase gene expression in the Syrian hamster. Endocrinology (2006) 147:4680–7. 10.1210/en.2006-0606 16873538

[34] Dardente H Klosen P Pévet P Masson-Pévet M. MT1 melatonin receptor mRNA expressing cells in the pars tuberalis of the European hamster: effect of photoperiod. J Neuroendocrinol (2003) 15:778–86. 10.1046/j.1365-2826.2003.01060.x 12834439

[35] Wittkowski W Bergmann M Hoffmann K Pera F. Photoperiod-dependent changes in TSH-like immunoreactivity of cells in the hypophysial pars tuberalis of the Djungarian hamster, *Phodopus sungorus*. Cell Tissue Res (1988) 251:183–7. 10.1007/BF00215463 3342436

[36] Böckers TM Niklowitz P Bockmann J Fauteck JD Wittkowski W Kreutz MR. Daily melatonin injections induce cytological changes in pars tuberalis-specific cells similar to short photoperiod. J Neuroendocrinol (1995) 7:607–13. 10.1111/j.1365-2826.1995.tb00798.x 8704734

[37] Hazlerigg DG Andersson H Johnston JD Lincoln G. Molecular characterization of the long-day response in the Soay sheep, a seasonal mammal. Curr Biol (2004) 14:334–9. 10.1016/j.cub.2004.01.057 14972686

[38] Dardente H Wyse CA Birnie MJ Dupré SM Loudon ASI Lincoln GA A molecular switch for photoperiod responsiveness in mammals. Curr Biol (2010) 20:2193–8. 10.1016/j.cub.2010.10.048 21129971

[39] Masumoto K-H Ukai-Tadenuma M Kasukawa T Nagano M Uno KD Tsujino K Acute induction of Eya3 by late-night light stimulation triggers TSHβ expression in photoperiodism. Curr Biol (2010) 20:2199–206. 10.1016/j.cub.2010.11.038 21129973

[40] Yasuo S Yoshimura T Ebihara S Korf H-W. Temporal dynamics of type 2 deiodinase expression after melatonin injections in Syrian hamsters. Endocrinology (2007) 148:4385–92. 10.1210/en.2007-0497 17540726

[41] Köhrle J. Local activation and inactivation of thyroid hormones: the deiodinase family. Mol Cell Endocrinol (1999) 151:103–19. 10.1016/S0303-7207(99)00040-4 10411325

[42] Watanabe M Yasuo S Watanabe T Yamamura T Nakao N Ebihara S Photoperiodic regulation of type 2 deiodinase gene in Djungarian hamster: possible homologies between avian and mammalian photoperiodic regulation of reproduction. Endocrinology (2004) 145:1546–9. 10.1210/en.2003-1593 14726436

[43] Hanon EA Routledge K Dardente H Masson-Pévet M Morgan PJ Hazlerigg DG. Effect of photoperiod on the thyroid-stimulating hormone neuroendocrine system in the European hamster (*Cricetus cricetus*). J Neuroendocrinol (2010) 22:51–5. 10.1111/j.1365-2826.2009.01937.x 19912472

[44] Barrett P Ebling FJP Schuhler S Wilson D Ross AW Warner A Hypothalamic thyroid hormone catabolism acts as a gatekeeper for the seasonal control of body weight and reproduction. Endocrinology (2007) 148:3608–17. 10.1210/en.2007-0316 17478556

[45] Saenz de Miera C Hanon EA Dardente H Birnie M Simonneaux V Lincoln GA Circannual variation in thyroid hormone deiodinases in a short-day breeder. J Neuroendocrinol (2013) 25:412–21. 10.1111/jne.12013 23282080

[46] Woitkewitsch AA Dependence of seasonal periodicity in gonadal changes on the thyroid gland in *Sturnus vulgaris* L. Dokl. Acad Sci URSS (1940) 27:741–5.

[47] Moenter SM Woodfill CJ Karsch FJ. Role of the thyroid gland in seasonal reproduction: thyroidectomy blocks seasonal suppression of reproductive neuroendocrine activity in ewes. Endocrinology (1991) 128:1337–44. 10.1210/endo-128-3-1337 1999155

[48] Dahl GE Evans NP Moenter SM Karsch FJ. The thyroid gland is required for reproductive neuroendocrine responses to photoperiod in the ewe. Endocrinology (1994) 135:10–5. 10.1210/endo.135.1.8013340 8013340

[49] Webster JR Moenter SM Barrell GK Lehman MN Karsch FJ. Role of the thyroid gland in seasonal reproduction. III. Thyroidectomy blocks seasonal suppression of gonadotropin-releasing hormone secretion in sheep. Endocrinology (1991) 129:1635–43. 10.1210/endo-129-3-1635 1874193

[50] Billings HJ Viguié C Karsch FJ Goodman RL Connors JM Anderson GM. Temporal requirements of thyroid hormones for seasonal changes in LH secretion. Endocrinology (2002) 143:2618–25. 10.1210/endo.143.7.8924 12072394

[51] Freeman DA Teubner BJW Smith CD Prendergast BJ. Exogenous T3 mimics long day lengths in Siberian hamsters. Am J Physiol Regul Integr Comp Physiol (2007) 292:R2368–72. 10.1152/ajpregu.00713.2006 17272662

[52] Murphy M Jethwa PH Warner A Barrett P Nilaweera KN Brameld JM Effects of manipulating hypothalamic triiodothyronine concentrations on seasonal body weight and torpor cycles in Siberian hamsters. Endocrinology (2012) 153:101–12. 10.1210/en.2011-1249 22028444

[53] Rodríguez EM Blázquez JL Pastor FE Peláez B Peña P Peruzzo B Hypothalamic tanycytes: a key component of brain-endocrine interaction. Int Rev Cytol (2005) 247:89–164. 10.1016/S0074-7696(05)47003-5 16344112

[54] Bolborea M Helfer G Ebling FJP Barrett P. Dual signal transduction pathways activated by TSH receptors in rat primary tanycyte cultures. J Mol Endocrinol (2015) 54:241–50. 10.1530/JME-14-0298 25878058

[55] Ono H Hoshino Y Yasuo S Watanabe M Nakane Y Murai A Involvement of thyrotropin in photoperiodic signal transduction in mice. Proc Natl Acad Sci U S A (2008) 105:18238–42. 10.1073/pnas.0808952105 19015516 PMC2587639

[56] Nakao N Ono H Yoshimura T Thyroid hormones and seasonal reproductive neuroendocrine interactions. Reproduction (2008) 136:1–8. 10.1530/REP-08-0041 18515309

[57] Dardente H. Melatonin-dependent timing of seasonal reproduction by the pars tuberalis: pivotal roles for long daylengths and thyroid hormones. J Neuroendocrinol (2012) 24:249–66. 10.1111/j.1365-2826.2011.02250.x 22070540

[58] Ikegami K Liao X-H Hoshino Y Ono H Ota W Ito Y Tissue-specific posttranslational modification allows functional targeting of thyrotropin. Cell Rep (2014) 9:801–10. 10.1016/j.celrep.2014.10.006 25437536 PMC4251493

[59] Henningsen JB Poirel VJ Mikkelsen JD Tsutsui K Simonneaux V Gauer F Sex differences in the photoperiodic regulation of RF-amide related peptide (RFRP) and its receptor GPR147 in the Syrian hamster. J Comp Neurol (2016) 524(9):1825–38. 10.1002/cne.23924 26518222

[60] Revel FG Saboureau M Masson-Pevet M Pevet P Mikkelsen JD Simonneaux V Kisspeptin mediates the photoperiodic control of reproduction in hamsters. Curr Biol (2006) 16:1730–5. 10.1016/j.cub.2006.07.025 16950111

[61] Wagner GC Johnston JD Clarke IJ Lincoln GA Hazlerigg DG. Redefining the limits of day length responsiveness in a seasonal mammal. Endocrinology (2008) 149:32–9. 10.1210/en.2007-0658 17901234

[62] Urbanski HF Doan A Pierce M. Immunocytochemical investigation of luteinizing hormone-releasing hormone neurons in Syrian hamsters maintained under long or short days. Biol Reprod (1991) 44:687–92. 10.1095/biolreprod44.4.687 2043739

[63] Barrell GK Moenter SM Caraty A Karsch FJ. Seasonal changes of gonadotropin-releasing hormone secretion in the ewe. Biol Reprod (1992) 46:1130–5. 10.1095/biolreprod46.6.1130 1391310

[64] Liu X Lee K Herbison AE. Kisspeptin excites gonadotropin-releasing hormone neurons through a phospholipase C/calcium-dependent pathway regulating multiple ion channels. Endocrinology (2008) 149:4605–14. 10.1210/en.2008-0321 18483150 PMC6116891

[65] Piet R de Croft S Liu X Herbison AE. Electrical properties of kisspeptin neurons and their regulation of GnRH neurons. Front Neuroendocrinol (2015) 36:15–27. 10.1016/j.yfrne.2014.05.006 24907402

[66] Ansel L Bolborea M Bentsen AH Klosen P Mikkelsen JD Simonneaux V. Differential regulation of kiss1 expression by melatonin and gonadal hormones in male and female Syrian hamsters. J Biol Rhythms (2010) 25:81–91. 10.1177/0748730410361918 20348459

[67] Smith JT Coolen LM Kriegsfeld LJ Sari IP Jaafarzadehshirazi MR Maltby M Variation in kisspeptin and RFamide-related peptide (RFRP) expression and terminal connections to gonadotropin-releasing hormone neurons in the brain: a novel medium for seasonal breeding in the sheep. Endocrinology (2008) 149:5770–82. 10.1210/en.2008-0581 18617612 PMC2584593

[68] Chalivoix S Bagnolini A Caraty A Cognié J Malpaux B Dufourny L. Effects of photoperiod on kisspeptin neuronal populations of the ewe diencephalon in connection with reproductive function. J Neuroendocrinol (2010) 22:110–8. 10.1111/j.1365-2826.2009.01939.x 20002963

[69] Ansel L Bentsen AH Ancel C Bolborea M Klosen P Mikkelsen JD Peripheral kisspeptin reverses short photoperiod-induced gonadal regression in Syrian hamsters by promoting GNRH release. Reproduction (2011) 142:417–25. 10.1530/REP-10-0313 21670127

[70] Caraty A Smith JT Lomet D Ben Saïd S Morrissey A Cognie J Kisspeptin synchronizes preovulatory surges in cyclical ewes and causes ovulation in seasonally acyclic ewes. Endocrinology (2007) 148:5258–67. 10.1210/en.2007-0554 17702853

[71] Sébert M-E Lomet D Saïd SB Monget P Briant C Scaramuzzi RJ Insights into the mechanism by which kisspeptin stimulates a preovulatory LH surge and ovulation in seasonally acyclic ewes: potential role of estradiol. Domest Anim Endocrinol (2010) 38:289–98. 10.1016/j.domaniend.2010.01.001 20097511

[72] Mason AO Greives TJ Scotti M-AL Levine J Frommeyer S Ketterson ED Suppression of kisspeptin expression and gonadotropic axis sensitivity following exposure to inhibitory day lengths in female Siberian hamsters. Horm Behav (2007) 52:492–8. 10.1016/j.yhbeh.2007.07.004 17706968 PMC2717891

[73] Greives TJ Humber SA Goldstein AN Scotti M-AL Demas GE Kriegsfeld LJ. Photoperiod and testosterone interact to drive seasonal changes in kisspeptin expression in Siberian hamsters (*Phodopus sungorus*). J Neuroendocrinol (2008) 20:1339–47. 10.1111/j.1365-2826.2008.01790.x 19094081 PMC2636859

[74] Saenz de Miera C Monecke S Bartzen-Sprauer J Laran-Chich MP Pevet P Hazlerigg DG A circannual clock drives expression of genes central for seasonal reproduction. Curr Biol (2014) 24:1500–6. 10.1016/j.cub.2014.05.024 24980500

[75] Simonneaux V Ancel C. RFRP neurons are critical gatekeepers for the photoperiodic control of reproduction. Front Endocrinol (2012) 3:168. 10.3389/fendo.2012.00168 23264769 PMC3524517

[76] Tsutsui K Saigoh E Ukena K Teranishi H Fujisawa Y Kikuchi M A novel avian hypothalamic peptide inhibiting gonadotropin release. Biochem Biophys Res Commun (2000) 275:661–7. 10.1006/bbrc.2000.3350 10964719

[77] Hinuma S Shintani Y Fukusumi S Iijima N Matsumoto Y Hosoya M New neuropeptides containing carboxy-terminal RFamide and their receptor in mammals. Nat Cell Biol (2000) 2:703–8. 10.1038/35036326 11025660

[78] Fukusumi S Habata Y Yoshida H Iijima N Kawamata Y Hosoya M Characteristics and distribution of endogenous RFamide-related peptide-1. Biochim Biophys Acta (2001) 1540:221–32. 10.1016/S0167-4889(01)00135-5 11583817

[79] Ukena K Iwakoshi E Minakata H Tsutsui K. A novel rat hypothalamic RFamide-related peptide identified by immunoaffinity chromatography and mass spectrometry. FEBS Lett (2002) 512:255–8. 10.1016/S0014-5793(02)02275-5 11852091

[80] Anderson GM Relf HL Rizwan MZ Evans JJ. Central and peripheral effects of RFamide-related peptide-3 on luteinizing hormone and prolactin secretion in rats. Endocrinology (2009) 150:1834–40. 10.1210/en.2008-1359 19022888

[81] Clarke IJ Sari IP Qi Y Smith JT Parkington HC Ubuka T Potent action of RFamide-related peptide-3 on pituitary gonadotropes indicative of a hypophysiotropic role in the negative regulation of gonadotropin secretion. Endocrinology (2008) 149:5811–21. 10.1210/en.2008-0575 18617613

[82] Ducret E Anderson GM Herbison AE. RFamide-related peptide-3, a mammalian gonadotropin-inhibitory hormone ortholog, regulates gonadotropin-releasing hormone neuron firing in the mouse. Endocrinology (2009) 150:2799–804. 10.1210/en.2008-1623 19131572

[83] Johnson MA Tsutsui K Fraley GS. Rat RFamide-related peptide-3 stimulates GH secretion, inhibits LH secretion, and has variable effects on sex behavior in the adult male rat. Horm Behav (2007) 51:171–80. 10.1016/j.yhbeh.2006.09.009 17113584 PMC1831848

[84] Kriegsfeld LJ Mei DF Bentley GE Ubuka T Mason AO Inoue K Identification and characterization of a gonadotropin-inhibitory system in the brains of mammals. Proc Natl Acad Sci U S A (2006) 103:2410–5. 10.1073/pnas.0511003103 16467147 PMC1413747

[85] Pineda R Garcia-Galiano D Sanchez-Garrido MA Romero M Ruiz-Pino F Aguilar E Characterization of the inhibitory roles of RFRP3, the mammalian ortholog of GnIH, in the control of gonadotropin secretion in the rat: in vivo and in vitro studies. Am J Physiol Endocrinol Metab (2010) 299:E39–46. 10.1152/ajpendo.00108.2010 20424142

[86] Leon S Garcia-Galiano D Ruiz-Pino F Barroso A Manfredi-Lozano M Romero-Ruiz A Physiological roles of gonadotropin-inhibitory hormone signaling in the control of mammalian reproductive axis: studies in the NPFF1 receptor null mouse. Endocrinology (2014) 155:2953–65. 10.1210/en.2014-1030 24823392

[87] Tsutsui K Ubuka T Son YL Bentley GE Kriegsfeld LJ. Contribution of GnIH research to the progress of reproductive neuroendocrinology. Front Endocrinol (2015) 6:179. 10.3389/fendo.2015.00179 26635728 PMC4655308

[88] Caraty A Blomenröhr M Vogel GMT Lomet D Briant C Beltramo M. RF9 powerfully stimulates gonadotrophin secretion in the ewe: evidence for a seasonal threshold of sensitivity. J Neuroendocrinol (2012) 24(5):725–36. 10.1111/j.1365-2826.2012.02283.x 22283564

[89] Decourt C Anger K Robert V Lomet D Bartzen-Sprauer J Caraty A No evidence that RFamide related peptide 3 directly modulates LH secretion in the ewe. Endocrinology (2016) 157(4):1566–75. 10.1210/en.2015-1854 26862995

[90] Rizwan MZ Poling MC Corr M Cornes PA Augustine RA Quennell JH RFamide-related peptide-3 receptor gene expression in GnRH and kisspeptin neurons and GnRH-dependent mechanism of action. Endocrinology (2012) 153:3770–9. 10.1210/en.2012-1133 22691552

[91] Poling MC Quennell JH Anderson GM Kauffman AS. Kisspeptin neurones do not directly signal to RFRP-3 neurones but RFRP-3 may directly modulate a subset of hypothalamic kisspeptin cells in mice. J Neuroendocrinol (2013) 25:876–86. 10.1111/jne.12084 23927071 PMC4022484

[92] Gouarderes C Mazarguil H Mollereau C Chartrel N Leprince J Vaudry H Functional differences between NPFF1 and NPFF2 receptor coupling: high intrinsic activities of RFamide-related peptides on stimulation of [35S]GTPgammaS binding. Neuropharmacology (2007) 52:376–86. 10.1016/j.neuropharm.2006.07.034 17011599

[93] Bonini JA Jones KA Adham N Forray C Artymyshyn R Durkin MM Identification and characterization of two G protein-coupled receptors for neuropeptide FF. J Biol Chem (2000) 275:39324–31. 10.1074/jbc.M004385200 11024015

[94] Mollereau C Mazarguil H Marcus D Quelven I Kotani M Lannoy V Pharmacological characterization of human NPFF(1) and NPFF(2) receptors expressed in CHO cells by using NPY Y(1) receptor antagonists. Eur J Pharmacol (2002) 451:245–56. 10.1016/S0014-2999(02)02224-0 12242085

[95] Gouarderes C Puget A Zajac JM. Detailed distribution of neuropeptide FF receptors (NPFF1 and NPFF2) in the rat, mouse, octodon, rabbit, guinea pig, and marmoset monkey brains: a comparative autoradiographic study. Synapse (2004) 51:249–69. 10.1002/syn.10305 14696013

[96] Dardente H Birnie M Lincoln GA Hazlerigg DG. RFamide-related peptide and its cognate receptor in the sheep: cDNA cloning, mRNA distribution in the hypothalamus and the effect of photoperiod. J Neuroendocrinol (2008) 20:1252–9. 10.1111/j.1365-2826.2008.01784.x 18752651

[97] Poling MC Kim J Dhamija S Kauffman AS. Development, sex steroid regulation, and phenotypic characterization of RFamide-related peptide (Rfrp) gene expression and RFamide receptors in the mouse hypothalamus. Endocrinology (2012) 153:1827–40. 10.1210/en.2011-2049 22355072 PMC3320244

[98] Ukena K Ubuka T Tsutsui K. Distribution of a novel avian gonadotropin-inhibitory hormone in the quail brain. Cell Tissue Res (2003) 312:73–9. 10.1007/s00441-003-0700-x 12712318

[99] Ubuka T Bentley GE Ukena K Wingfield JC Tsutsui K. Melatonin induces the expression of gonadotropin-inhibitory hormone in the avian brain. Proc Natl Acad Sci U S A (2005) 102:3052–7. 10.1073/pnas.0403840102 15708982 PMC549437

[100] Chowdhury VS Yamamoto K Ubuka T Bentley GE Hattori A Tsutsui K. Melatonin stimulates the release of gonadotropin-inhibitory hormone by the avian hypothalamus. Endocrinology (2010) 151:271–80. 10.1210/en.2009-0908 19952272

[101] Bentley GE Perfito N Ukena K Tsutsui K Wingfield JC. Gonadotropin-inhibitory peptide in song sparrows (*Melospiza melodia*) in different reproductive conditions, and in house sparrows (*Passer domesticus*) relative to chicken-gonadotropin-releasing hormone. J Neuroendocrinol (2003) 15:794–802. 10.1046/j.1365-2826.2003.01062.x 12834441

[102] Perfito N Zann R Ubuka T Bentley G Hau M. Potential roles for GNIH and GNRH-II in reproductive axis regulation of an opportunistically breeding songbird. Gen Comp Endocrinol (2011) 173:20–6. 10.1016/j.ygcen.2011.04.016 21536042

[103] Revel FG Saboureau M Pevet P Simonneaux V Mikkelsen JD. RFamide-related peptide gene is a melatonin-driven photoperiodic gene. Endocrinology (2008) 149:902–12. 10.1210/en.2007-0848 18079200

[104] Janati A Talbi R Klosen P Mikkelsen JD Magoul R Simonneaux V Distribution and seasonal variation in hypothalamic RF-amide peptides in a semi-desert rodent, the jerboa. J Neuroendocrinol (2013) 25:402–11. 10.1111/jne.12015 23289624

[105] Simonneaux V Ansel L Revel FG Klosen P Pévet P Mikkelsen JD. Kisspeptin and the seasonal control of reproduction in hamsters. Peptides (2009) 30:146–53. 10.1016/j.peptides.2008.06.006 18619505

[106] Greives TJ Mason AO Scotti MA Levine J Ketterson ED Kriegsfeld LJ Environmental control of kisspeptin: implications for seasonal reproduction. Endocrinology (2007) 148:1158–66. 10.1210/en.2006-1249 17185375

[107] Clarke IJ Smith JT Henry BA Oldfield BJ Stefanidis A Millar RP Gonadotropin-inhibitory hormone is a hypothalamic peptide that provides a molecular switch between reproduction and feeding. Neuroendocrinology (2012) 95:305–16. 10.1159/000332822 22286004

[108] Sari IP Rao A Smith JT Tilbrook AJ Clarke IJ. Effect of RF-amide-related peptide-3 on luteinizing hormone and follicle-stimulating hormone synthesis and secretion in ovine pituitary gonadotropes. Endocrinology (2009) 150(12):5549–56. 10.1159/000332822 19808777

[109] Smith JT Young IR Veldhuis JD Clarke IJ. Gonadotropin-inhibitory hormone (GnIH) secretion into the ovine hypophyseal portal system. Endocrinology (2012) 153(7):3368–75. 10.1210/en.2012-1088 22549225 PMC3380300

[110] Jorgensen SR Andersen MD Overgaard A Mikkelsen JD. Changes in RFamide-related peptide-1 (RFRP-1)-immunoreactivity during postnatal development and the estrous cycle. Endocrinology (2014) 155:4402–10. 10.1210/en.2014-1274 25144921

[111] Smith JT Popa SM Clifton DK Hoffman GE Steiner RA. Kiss1 neurons in the forebrain as central processors for generating the preovulatory luteinizing hormone surge. J Neurosci (2006) 26:6687–94. 10.1523/JNEUROSCI.1618-06.2006 16793876 PMC6673844

[112] Simonneaux V Bahougne T. A multi-oscillatory circadian system times female reproduction. Front Endocrinol (2015) 6:157. 10.3389/fendo.2015.00157 26539161 PMC4611855

[113] Gibson EM Humber SA Jain S Williams WP III Zhao S Bentley GE Alterations in RFamide-related peptide expression are coordinated with the preovulatory luteinizing hormone surge. Endocrinology (2008) 149:4958–69. 10.1210/en.2008-0316 18566114 PMC2582915

[114] Russo KA La JL Stephens SB Poling MC Padgaonkar NA Jennings KJ Circadian control of the female reproductive axis through gated responsiveness of the RFRP-3 system to VIP signaling. Endocrinology (2015) 156:2608–18. 10.1210/en.2014-1762 25872006 PMC4475714

